# Physical activity promotion in daily exercise therapy: the perspectives of exercise therapists in German rehabilitation settings

**DOI:** 10.1186/s13102-019-0143-7

**Published:** 2019-12-02

**Authors:** Wolfgang Geidl, Judith Wais, Cheyenne Fangmann, Ewnet Demisse, Klaus Pfeifer, Gorden Sudeck

**Affiliations:** 1 Department of Sport Science and Sport, Division of Exercise and Health, Friedrich-Alexander University Erlangen-Nürnberg, Gebbertstraße 123b, 91058 Erlangen, Germany; 2 Institute of Sport Science, Department of Education and Health Research, Eberhard Karls University Tübingen, Wächterstraße 76, 72074 Tübingen, Germany

**Keywords:** Exercise, Behavioural change, Motor behaviour, Physical therapy

## Abstract

**Background:**

This study aims to explore exercise therapists’ perspectives on the topic of physical activity promotion (PAP) with a focus on identifying (i) the intervention content and methodological approaches used for promoting physical activity (PA) in daily practice and (ii) the barriers and facilitators that affect PAP.

**Methods:**

This qualitative study comprised the heads of exercise therapy departments (*n* = 58; 41% women; mean age = 45 years) from different rehabilitation clinics in Germany. Each participant took part in a semi-structured focus-group discussion on PAP in exercise therapy. The findings of the focus groups were processed and interpreted using a conventional qualitative content analysis.

**Results:**

The exercise therapists demonstrated detailed didactic–methodological strategies and action orientations for PAP. The identified core topics of the content and methods of PAP were (1) conceptualization, (2) exercise and PA for enjoyment and pleasure, (3) education with practice–theory combinations, (4) media and materials for self-directed training, and (5) strategies to enhance personal responsibility and independence. The core topics for the associated barriers and facilitators were (1) structural conditions, (2) the role of exercise therapists, (3) the interdisciplinary rehabilitation team, (4) rehabilitant experiences and expectations, and (5) aftercare services.

**Conclusion:**

The topic of PAP is addressed with a high level of variability; exercise therapists involved in this study identify various methods and content for the promotion of PA within their individual practices. However, they display a limited awareness of existing evidence- and theory-based concepts for the promotion of PA as well as underlying theories of behavioural change. This variability may be due to the lack of a defined common framework for promoting PA, insufficient emphasis being placed on PA promotion in the current curricula and training, or extensive conceptual differences within German exercise therapy departments (e.g. different weighting of PAP).

## Background

Physical activity promotion (PAP) is a central goal of exercise therapy [1, 2]. However, raising one’s activity levels is rather difficult for most clients. For example, exercise training (e.g. endurance or strengthening exercises) is the most common strategy used to modify physical activity (PA) levels in people with a chronic obstructive pulmonary disease, but their average increases in PA levels remain low nonetheless at roughly 10% [3]. In addition, this systematic review from Mantoani et al. [3] elicits that seven out of 21 exercise studies showed no substantial increases in PA in persons with a chronic obstructive pulmonary disease. Long-term adherence to PA after exercise-based rehabilitation is typically also poor for other health conditions, including low back pain [4] and cardiovascular disease [5, 6]. Morris and Jenkins [7] conclude that therapists often fail to achieve and maintain significant increases in PA with their clients.

The limited effects of exercise therapy in promoting PA have prompted the development of concepts of theory-based exercise therapy interventions. These approaches are characterised by the use of educational strategies, and the implementation of adequate exercise content in combination with various behavioural change techniques [8–11]. Thus, these approaches differ on the one hand in *what* they do (didactical approach) and on the other hand in *how* they implement it (methodological approach). These new didactical–methodological approaches address improvements in the functions of the body and clients’ relevant personal factors regarding PA behaviour – namely, their attitudes, skills, emotions, beliefs, and knowledge [12]. Thus, these approaches enhance the likelihood of successfully increasing clients’ PA levels [13].

However, the manner in which these new concepts are disseminated and implemented in the practice of exercise therapy remains uncertain. In Australia, a cross-sectional survey involving 216 physiotherapists reported a high number of respondents (85.1%) who stated that they promoted PA beyond the therapeutic treatment (so called non-treatment PA) often or all of the time [14]. The respondents indicated their use of 29 different behavioural change techniques to either promote non-treatment PA or encourage adherence to rehabilitation exercise [15]. In contrast, Freene et al. [16] report that only half of Australian physiotherapists regularly use interventions for PAP. In this context, several critical findings regarding the dissemination and implementation of PAP have been discussed. A scoping review conducted by Lowe et al. [17] shows that despite PAP being an increasing area of interest, within physical therapy, it remains a rather undeveloped field of research. A qualitative study involving 12 physiotherapists in the UK identified inconsistencies and a general lack of guidance within the practice in regards to PAP, which largely focused on the short-term restoration of bodily functions [18]. Another study conducted by Lowe et al. [19] concluded that in physical therapy practice PA levels are not routinely assessed and that brief interventions to enhance PA levels are not routinely delivered. This is supported by Williams et al. [20], who conclude that physiotherapists value PAP but that goal-directed PAP remains largely absent from their practice. Furthermore, Morris and Jenkins [7] suggest that physical therapy professionals may be practicing health promotion, including the promotion of a physically active lifestyle, in an inadequate and inconsistent way.

In the context of Germany, PAP is defined as a central goal of exercise therapy within the framework of rehabilitation [21]. Our previously conducted national survey of exercise therapy practice in this setting reveals that exercise therapists consider PAP an important goal [22] and that they consistently regard various behavioural change techniques as important aspects of the exercise therapy they conduct with clients. However, the dissemination of theoretically sound evidence-based concepts for PAP has reached only half of exercise therapy departments in Germany [23], and the use of such concepts is quite heterogeneous. Furthermore, there is little research into the factors that influence PAP in the practice of daily exercise therapy. Thus, in promoting PA on an individual level, the concrete content and methods used by German exercises therapists and the extent to which they are implemented have yet to be thoroughly investigated. As such, this study aims to explore the views of exercise therapists on PAP and identify 1) the didactical–methodological approaches that exercise therapists use with the aim of promoting PA and 2) the facilitators and barriers that affect PAP. The identification of these factors forms the basis for the targeted optimisation of PAP within daily exercise therapy practice.

## Methods

This study is part of a larger project entitled ‘Exercise therapy in medical rehabilitation: A survey at facility and practitioner level.’ Quantitative and qualitative methods were integrated in the two consecutive phases of this project. This study uses data from the second qualitative phase of the project. We have based our reporting on the checklist of the consolidated criteria for reporting qualitative studies (COREQ) [24]. A detailed description of the project can be found in the study protocol by Geidl et al. [25].

The quantitative data from the first phase of the project have already been published [22, 23, 26]. Thus, the first phase is only briefly described here, as the participants of the present study were recruited from the sample who participated in the first phase. There, the heads of different exercise therapy departments were invited to participate in a standardised and quantitative written survey. The aim was to compile a comprehensive national overview of conceptual and process-related features of exercise therapy at the level of individual rehabilitation facilities. Based on this quantitative survey, the second phase of the project assessed the subjective views of exercise therapists in a more detailed and descriptive manner. For this purpose, two one-and-a-half day workshops were held. Fifty-eight heads of exercise therapy departments from different facilities held discussions on various topics of exercise therapy. In Germany, exercise therapy is offered in disease-specific rehabilitation clinics. Seeing as though there may be peculiarities in the topic of PAP in people with different health conditions, the discussions were conducted in six disease-specific focus groups.

### Design and implementation

As part of the two workshops, we conducted semi-structured focus-group discussions with the exercise therapists in April 2016. The discussions were held in the conference center of a local sport federation (Landessportbund Hessen) in Frankfurt/Main, Germany on each of the following six health conditions: orthopaedics (back pain, total hip/knee replacement), neurology, oncology, psychosomatics, and addiction. The focus group discussions lasted on average 130 min (Range 116–141 min) resulting in 12 h 59 min of audio-recorded discussion. The discussions were moderated by three scientific project workers (two of them male) with a scientific degree in sport science and expertise in the practice of exercise therapy. Because of the close affiliation the moderators had to the research project, major emphasis was placed in advance on training and a pre-test in order to ensure the moderators were open to the emergence of new topics and to practice their handling of extreme cases and unexpected situations.

The results presented here are based on the following central question: ‘What elements within your exercise therapy practice contribute to rehabilitants’ PA adherence after their inpatient rehabilitation?’. This question was raised after a short introduction ‘A central goal of rehabilitation is the initiation or maintenance of physically active lifestyles. There are different approaches used to pursue this goal.’ This central question is based on the rehabilitation goals formulated within the framework of rehabilitation [21] and was elaborated on through discussion by the entire research team. During the focus group discussions, no non-participants were present in the room.

### Sample

Exercise therapy heads who had previously taken part in the nationwide survey were contacted by post. The invited individuals came from clinics which had been attributed to six typical concepts of exercise therapy that differed regarding the role of PAP [23]. In a two-phase invitation process, a total of 166 invitations were sent out, resulting in 73 registrations (response rate = 44%). Each workshop took place with three disease-specific subgroups (*n* = 8–12). The participants of the workshops came from all over Germany and most of them neither knew each other nor the moderators before their focus group discussion. Table 1 provides an overview of the characteristics of the 58 participants.

**Table 1:** Characteristics of the participants (focus groups, *n* = 58). – -

	Total		Orthopaedics (back pain)		Orthopaedics (THR, TKR)*		Neurology		Oncology		Addiction		Psychosomatics	
	n	M (Range SD)	n	M (Range SD)	n	M (Range SD)	n	M (Range SD)	n	M (Range SD)	n	M (Range SD)	n	M (Range SD)
*Characteristic*														
Number of participants	58		11		10		9		8		10		10	
Form of care^a^														
Inpatient	47		5		8		9		7		9		9	
Outpatient	29		9		8		3		2		1		6	
Age (in years)		45 (28–1 ± 10)		44 (28–58 ± 12)		50 (39–57 ± 7)		45 (34–53 ± 6)		44 (28–56 ± 11)		45 (28–58 ± 10)		43 (30–60 ± 10)
Gender														
Female	24		6		5		1		4		4		4	
Male	34		5		5		8		4		6		6	
Head of exercise therapy department														
Yes	50		8		9		9		8		8		8	
No^b^	8		3		1						2		2	
Educational background														
Physiotherapy	22		4		6		5		3		1		3	
Exercise therapy^c^ / sport science	38		8		5		5		5		8		7	
Other	12		1		4		2		–		3		2	
Professional experience (in years)														
Educational background in physiotherapy		21 (4–35 ± 9)		24 (8–35 ± 12)		22 (18–31 ± 5)		23 (19–28 ± 3)		26 (18–34 ± 8)		5–		13 (4–24 ± 10)
Educational background in exercise therapy / sport science		17 (2–39 ± 10)		18 (3–39 ± 13)		19 (2–35 ± 14)		14 (5–22 ± 8)		11 (2–28 ± 12)		18 (6–31 ± 8)		19 (10–34 ± 9)

*Notes*. (THR, TKR)* = total hip replacement, total knee replacement; ^a^multiple answers possible; ^b^were sent to represent the head of the department; ^c^in Germany, this refers to the education of *Sport und Bewegungstherapie*

### Data sources and data analysis

The focus-group discussions were processed and interpreted using the conventional qualitative content analysis defined by Hsieh et al. [27] and described in detail by Kuckartz [28]. Following the word-for-word transcription of all focus-group interviews, the content of the transcripts was encoded using MAXQDA (Version 12) software. The main categories (for content and methods as well as for barriers and facilitators) were formed by five persons (WG, JW, CF, KP and GS) after initiating the textual work which was carried out by two persons (JW, CF) in consultation with the other authors. In accordance with the encoding rules, two independent actors (JW, CF) allocated the text material to these main categories. The sub-categories were then determined inductively, and all of the according text material was allocated to each category by one author (JW or CF). To assess the quality of the sub-category system, 30% of the text material (two disease-specific groups) was encoded by a second independent actor (JW or CF). The calculated kappa coefficients between 0.67 (orthopaedics [total hip/knee replacement]) and 0.85 (orthopaedics [back pain]) indicated a good to very good intercoder agreement [29]. The final step involved holding iterative discussions with the research team. The overriding goal of these final discussions was to obtain as broad a view as possible on the topic of PAP in exercise therapy. For this purpose, we first presented a summary of the results for each disease-specific group and identified the general sub-categories of the content. In a second step, differences and similarities between the disease-specific groups were also discussed in the research team, and the special features that existed from our expert point of view were worked out in the results section.

## Results

The results section is divided into two parts. Part 1 lists the identified main categories of the content and methods of PAP reported by exercise therapists. Part 2 provides a description of the associated perceived barriers and enabling factors of PAP. Table 2 and Table 3 provide an overview of the main topics of both these parts. Throughout the results section, topics are primarily discussed across diseases and explained with examples from individual disease-specific groups. If we identified significant differences or similarities in the disease-specific group discussions, it is pointed out separately.

**Table 2:** Main categories of the focus-group discussion on content and methods of physical activity promotion

(1) Explicit concept-based approaches in exercise therapy are comprised of:	
- volitional support (high concept-base) vs. movement experience (low concept-base); and	
- institution-specific adaptations.	
(2) The action pattern of ‘Fun and joy in exercise and physical activity’ includes:	
- a diverse spectrum of exercise interventions;	
- (the rediscovery of) individually tailored and enjoyable activity;	
- the promotion of group experiences and activities;	
- reflections on exercise experiences; and	
- an effort to make rehabilitants more receptive to positive and joyful exercise experiences.	
(3) Knowledge transfer to the rehabilitants as a theory–practice combination involves:	
- a central principle of reflection in the pairing of knowledge and exercise experience; and	
- the demonstration of knowledge through proximity to everyday life and pictorial language.	
(4) Use of material and media for independent training and practice involves:	
- the common but mostly ineffective use of materials in paper form; however,	
- the search continues for modern forms of media use.	
(5) Strategies to promote personal responsibility include:	
- the independent use of therapy-free time fostered by different elements;	
- a reduction in consuming attitudes of rehabilitants; and	
- preparation and strategies for concrete continuation at the place of residence of the rehabilitants.

**Table 3:** Main categories of the focus-group discussion on the barriers to and facilitators of physical activity promotion

(1) Individuality vs. organisational–structural conditions	
- Guidelines and standards impair patient-centred care (barrier).	
- A large facility may offer a wide range of different exercise therapies (facilitator), while a small facility may have a family atmosphere and potential for a significant therapist–patient to develop (facilitator).	
- The changing of therapists may be quite frequent (barrier).	
(2) The role of exercise therapists	
- They have empathy for the needs of rehabilitants (facilitator).	
- They can be persuasive with a view to promoting PA (facilitator).	
(3) Cooperation, communication, and common messages in the interdisciplinary rehabilitation team	
- Joint messages promote PA (facilitator).	
- Team exchange compensates for a lack of consistency in therapists (facilitator).	
- The medical dominance within therapy prescription partially impairs the suitability of exercise plans (barrier).	
(4) Expectations and previous exercise experiences of rehabilitants	
- Rehabilitants expect passive interventions such as massages (barrier).	
- Rehabilitants can motivate themselves based on their previous experience of exercise (facilitator).	
- The older rehabilitants are less motivated (barrier).	
(5) Quantity and quality of rehabilitation aftercare services	
- There is a possibility of continuing aftercare in the same facility (facilitator).	
- It is important to ensure the quality of aftercare services (facilitator/barrier).	
- Some aftercare actors offer follow-up contacts (facilitator).	

### Main categories of content and methods of physical activity promotion

#### (1) Extent of explicit concept-based approaches in exercise therapy

Concept-based approaches are characterised as planned and structured procedures. This also includes the use of externally developed, standardised intervention programmes, e.g. the MoVo-Lisa-programme [30] in which different therapy hours build on each other and the individual therapy hours are described in detail in a manual.

The focus groups demonstrated that concept-based approaches with varying degrees of differentiation are used within exercise therapy. A rather differentiated concept-based approach was found in connection with the use of behavioural volitional techniques (e.g. the elaboration of action- and coping-plans), which were frequently raised in the focus-group discussions. In the focus groups, orthopaedics (back pain), addiction, psychosomatics, and oncology, a motivational-volitional PAP concept from Germany, known as the ‘MoVo-LISA concept’ [30], were discussed:

There’s a good programme called MoVo-LISA, we’ve changed it a bit, adapted it to leisure in general […] Most people say, I actually want to do something, but to plan the whole thing correctly we must ask: ‘What are you doing? When will you do it? Who are you doing it with? Do you have enough options? Does it suit you? Does it fit in with your lifestyle?’ (Addiction #00:47:53–6#)

It is noticeable here that in many exercise therapy departments, specific intervention components of existing PAP programmes are used without needing to implement the entire programme. As well as this, the selected intervention components are not typically implemented as originally developed but adapted to the exercise therapists’ expertise and the structural conditions of the department.

#### (2) Action pattern: ‘Fun and joy in exercise and physical activity’

Across all health conditions, there was a consensus that conveying ‘fun and joy in exercise and PA’ is an important aspect of PA promotion. This was regarded as a basic prerequisite for the long-term maintenance of a physically active lifestyle by many of the focus-group participants.

Within numerous and multifaceted statements on this subject, a certain pattern was often discernible: Rehabilitation facilities try to offer a wide range of exercise and sport activities. This wide range makes it possible to effectively respond to the needs of each individual by taking existing personal preferences into account and allowing said individual to experience new PA stimuli. One exercise therapist described this connection from her point of view:

We have a relatively broad spectrum of different exercise therapies. We have 20 different sport groups […]. From archery, stick fighting, juggling, trampoline, movement meditation, etc. […] So, we have a lot of things, and in archery there are often statements like, ‘Whoa, that’s great, I want to do that at home and where can I do that?’ Not everything is for everyone, but I just have to look. And the patients must also be allowed to decide for themselves: ‘What do I enjoy? What gives me pleasure? And where can I stay consistent? Because only when I have fun with it will I stay with it in the long run.’ (Neurology #00:57:56–3#)

Another important intervention element for the action pattern of ‘fun and joy’ is the experiences found within group exercise settings. Groups play an important supportive role, thus aiding the promotion of and adherence to exercise and PA:

But when you find something in a circle of friends where you can do sports together and its fun, that’s the most sustainable thing in my eyes that you can give people. […] The long-term motivation to do exercise and stay in it is not the knowledge, […] but rather the fun in the group, the sociability, the common sports experience in the group. (Orthopaedics [back pain] #00:07:29–8#)

In order to convey fun and joy in exercise and PA, the importance of reflecting on the experience of movement was also noted. Methods to promote this reflection were discussed in the focus groups.

#### (3) Knowledge transfer to rehabilitants as a theory–practice combination

The two main topics of knowledge transfer are 1) knowledge about the effects of PA (i.e. effect knowledge) and 2) knowledge about the execution of PA (i.e. action knowledge) (compare [31]). A central theme in the field of knowledge transfer was the pairing of practical exercise experience with reflection. For example, this was discussed in the orthopaedics (back pain) focus group:

Only patient education, pure lecture is useless. So, we exercise therapists live from the fact that we don’t just give lectures but connect them with exercises and practice. (Orthopaedics [back pain] #00:14:33–4#)

Teachings about the knowledge of PA effects were addressed in four of the six focus groups and were related to the physical and psychological effects of PA. Exercise therapists described their use of a three-step method for outlining the short-term effects by a) targeting the possible effects of exercise; b) questioning/assessing the effects during and after the exercise; and c) reflecting on these effects in a subsequent conversation.

Linking theory with practice and reflecting on exercise experiences were two principles that were also applied to the field of imparting action knowledge. For example, methods for teaching self-directed load control (e.g. the correct independent control of the training intensity with the help of a heart rate monitor) were theoretically prepared, tested in practice, and then reflected upon in the group.

Some exercise therapists try to prepare rehabilitants for the continuation of exercise and PA after their rehabilitation. To address this, they discussed suitable exercises, physical activities, and possibilities for daily PA integration, and they provided general information as well as personal counselling to the rehabilitants. In summary, from a practitioner’s perspective, sustainable knowledge transfer is best achieved through the connection and combination of movement practice and reflection.

#### (4) Use of material and media for independent training and practice

The widespread use and efficiency of materials used for physical training and practice were often discussed:

But I always question how meaningful these exercise sheets are. In my experience, if patients want sheets right from day one, they forget them or throw them away. (Orthopaedics [total hip/knee replacement] #00:33:43–6#)

The most extensive discussion on this topic of material and media use took place in the orthopaedics (total hip/knee replacement) focus group:

[…] what kind of sheets do the patients get, training books, etc. and then there are also sceptical therapists who claim, ‘He takes it and throws it right into the trash can.’ So, you think about which format is the right one […]. (Neurology #00:35:07–8#)

As a solution, the need to reconsider the use of conventional print materials became increasingly visible. Moving away from paper and towards the use of more modern formats, such as smart-phone applications, photographs, video recordings of the exercises on the rehabilitants’ smartphones, or computer-aided training and exercise programmes, may be more effective.

#### (5) Strategies to promote personal responsibility

Strategies to promote the independent execution of exercises were frequently discussed. In one clinic, for example, the rehabilitants were required to perform independent PA and exercise during their therapy-free (leisure) time:

We make sure that the patients not only become active in the therapy programme, but also organise something movement-related themselves, become jointly active, in addition to the therapy, in their therapy-free time. […] And we’ve actually had quite good experiences with that […] they simply take more responsibility for themselves […]. (Psychosomatics #00:11:38–8#)

The exercise therapists were critical of the fact that the ‘consuming’ role of clients had to be reduced and that active, independent action had to be initiated and promoted:

We are definitely bringing the patients to the point […] where personal responsibility is much more important than just the therapeutic intervention […]. (Neurology #00:32:45–6#)

In some focus groups, it was reported that after prior instruction, rehabilitants were able to use the training rooms independently at times when there was no need for therapy.

### Barriers and facilitators for implementing content and methods of physical activity promotion

The second part of the results section presents the core topics regarding the barriers to and facilitators of successful PAP. The core topics are often closely related to the main topics of part 1 of the results section.

#### (1) Individuality vs. organisational–structural conditions

In all focus groups, adequate organisational and structural conditions were viewed as important prerequisites for successful PAP. However, some exercise therapists stated that ‘fun and joy in exercise and physical activity’ are difficult to achieve due to several factors. One of these obstructing factors is the so called therapy standards. Therapy standards are specified by the German pension insurance for quality assurance purposes. The requirements for these standards are sometimes perceived as excessively rigid prescriptions that restrict the possibility of individually tailored therapy:

I think the main problem with these therapy specifications is that we have to report them using the KTL [Klassifikation Therapeutischer Leistungen; Translation: classification of therapeutic interventions] and the requirements that we have to meet lead to a lack of individuality. (Oncology #00:43:24–8#)

In all focus groups, the role of the clinic size was controversially discussed with regard to individuality. Small rehabilitation facilities have the advantage of close contact with rehabilitants, and the atmosphere is perceived as being more familiar. But for smaller facilities, it is often not possible to offer a wide range of exercise therapies. Large rehabilitation facilities can offer a wide range of exercise experiences to ensure that rehabilitants are more likely to have the option of making an individually tailored choice.

And the bigger a facility […] the bigger the range of exercise therapy offers. We have […] a small, familiar facility. […] After a week, I know every patient. […]. But I can’t offer as much as I can in a bigger clinic. (Orthopaedics [back pain] #00:35:04–7#)

The organisational form of the therapy was identified as a crucial factor to ensure individuality and patient-orientation. There was a broad consensus that an individual approach could be implemented in a one-on-one therapy situation. In group therapies, however, the heterogeneity of the rehabilitants and the changing composition of the (open) groups prevent an individual approach. Rehabilitants are often over- or under-challenged in groups, and this impairs a positive exercise experience. In addition, the need to attain consistency among therapists is clearly evident but not always achieved due to a lack of time, personnel resources, and planning difficulties.

So, it may be that a patient has an admission, an initial assessment by a physiotherapist. And from the next treatment on, the therapist changes. Then he has three, four, five therapists. […] the one-on-one treatment in our facility is very decimated anyway, […] simply because the capacity is not there. […] Everything in our facility is also very strongly determined by the planning department. So, the planning is above everything. […] And the therapeutic goals only play a small role. (Orthopaedics [back pain] #00:22:01–0#)

In summary, adequate organisational and structural framework conditions are seen as important prerequisites for sustainable PAP. It often seems impossible to consider the individual factors of the rehabilitants, resulting in the over- or under-training of these individuals.

#### (2) The role of exercise therapists

The focus groups also addressed the role of therapists in social situations within exercise therapy. The need to establish a positive and close relationship with the rehabilitants was thoroughly discussed.

Well, I have another aspect that goes beyond the content […] to give the patient the feeling that he is constantly seen. This means that, with this therapist relationship, he always has someone to whom he can turn to, but then he also has the feeling that he is perceived by what he does, how he behaves, what he shows of himself. (Psychosomatics #00:56:11–0#)

Empathy and authenticity in one’s own therapeutic practice were particularly emphasised. These characteristics were seen as facilitators in promoting a trusting and personal bond between rehabilitants and therapists.

It was also considered important for exercise therapists to be able to perceive and respond to the needs of rehabilitants. The ability to adapt the exercise therapy programme to the respective requirements of the rehabilitants was regarded as a facilitating factor. With these adaptations, the action pattern of ‘fun and joy in exercise and physical activity’ can be better served.

In a nutshell, the empathy, authenticity, and responsiveness of the therapists and their methods are considered important for PAP.

#### (3) Cooperation, communication, and common messages in the interdisciplinary rehabilitation team

Cooperation and transparent communication across occupational groups in the interdisciplinary rehabilitation team were deemed beneficial for PAP. Furthermore, a lively exchange of information about the rehabilitants was identified as an important factor. The importance of all therapeutic actors attaining a common and uniform language for the rehabilitants was also emphasised many times:

We have team meetings twice a week, all disciplines are there. And then, of course, it’s comparatively easy in this context to respond very individually to the patients and to find a common language. […] I have also had the experience that the patients already notice when the team of therapists works and speaks one language. (Orthopaedics [back pain] #00:06:16–4#)

In all health conditions, a hierarchical structure within the multi-professional team was seen as an obstacle to the promotion of PA. Medical dominance, lack of information, or low levels of interest among doctors in exercise therapy were described as the reasons for frequent misdirection.

Well, we have that problem over and over again. It’s simply also about the fact that doctors prescribe things, simply prescribe prescriptions and without actually knowing what we actually do in the therapies. (Oncology #00:26:19–2#)

To conclude, transparent communication and cooperation between occupational groups are crucial to attain optimal PAP. In addition to this, the exchange of information within a therapy team allows for greater consistency in the delivery of treatment plans, irrespective of any changes of therapists.

#### (4) Expectations and previous exercise experience of rehabilitants

Exercise therapists estimate experiences and expectations of the rehabilitants to have a great influence on the independent initiation and maintenance of PA. Our participants discuss them both as beneficial and obstructive factors. In particular, the expectation of passive therapies (e.g. massage) during rehabilitation is increasingly addressed as an obstacle of PAP in all chronic conditions. The expectation of treatment instead of attaining an active role in the rehabilitation process was discussed.

Yes, and we all know the patients who arrive and say, ‘I’ll come to the application now’ and would like to lie down somewhere and then ‘just do it’ today. And that’s very important, that you say from the beginning, ‘No, my dear patient, that’s not how it is. This is rehabilitation, not a cure. Move it!’ (Neurology #00:29:45–5#)

The self-motivation of the rehabilitants based on their previous exercise experience was perceived as a beneficial factor. Rehabilitants who had positive exercise experiences in the past were linked to pursuing more long-term activities:

We now often have patients who already have some previous sporting experience. It’s easier there, of course. They may have lost it over the years: […] they couldn’t do as many activities for themselves anymore because of family, job, and so on. But the basis is there, you can build somewhere on it. (Oncology #00:47:58–5#)

In addition to these more motivational characteristics, the age of the rehabilitants was discussed and almost exclusively addressed as a barrier. It is more difficult for older people to impart adequate physical activity for the time after rehabilitation.

Briefly summarised, the expectations of many rehabilitants to *undergo* therapy are mentioned as barriers to the successful promotion of PA. The age of the rehabilitants is also seen as an aggravating factor if they are older.

#### (5) Quantity and quality of rehabilitation aftercare services

The final core topic discusses the beneficial and impeding aspects of rehabilitation aftercare services. A sufficient number of high-quality aftercare services is considered important for a supply close to home of the rehabilitants. These aftercare services are often associated with the rehabilitation facility and regarded as a beneficial factor. The rehabilitants are already familiar with the conditions and staff of the facility, therefore eliminating any inhibitions that would otherwise limit activities in an unknown facility. One participant described this as follows:

The people who get excited come to us for the most part because they feel comfortable with us and know the processes, the therapists, the equipment. (Orthopaedics [total hip/knee replacement] #00:23:19–0#)

The existence of aftercare services at the place of residence of the rehabilitants was discussed rather controversially. Some stressed the fact that there are nationwide aftercare services already available, while some complained that the accessibility of aftercare services may be restricted depending on the place of residence of the rehabilitants.

This controversial discussion also raised scepticism regarding the quality of existing aftercare services. The question was raised as to whether the aftercare services adequately address the respective health problems of the rehabilitants. It was therefore suggested that rehabilitation facilities should provide reports on rehabilitants health status for continuing rehabilitation providers.

To sum up, an adequate number of high-quality aftercare services is essential. Services affiliated with the facility where the original treatment took place are seen as beneficial. Although a large number of aftercare services are already available, the accessibility of these facilities and thus, their effectiveness, are questioned. Furthermore, the quality of services administered at the place of residence of the rehabilitants also raises concerns and scepticism.

## Discussion

Through this study, comprehensive knowledge was acquired regarding exercise therapists’ unique perspectives and beliefs in relation to PAP. We especially identified the content and methods used for promoting PA in daily exercise therapy practice and the associated perceived barriers and facilitators. The study expands our understanding of the topic of PAP from a practitioner’s point of view and explores the needs, challenges, and possibilities in the field of PAP. The expert knowledge and attitudes of the exercise therapy practitioners are important, as they translate scientific evidence into practice [32]. Thus, our results form the basis for systematic improvement and implementation of high-quality PAP in exercise therapy.

In line with previous studies in which physical therapists indicated the use of different behavioural change techniques and interventions to promote PA [15, 17, 19, 33], the 58 leading exercise therapists in our study name a multitude of different methods and content for PAP in their everyday practice. In the topics mentioned, there are clear references to the pedagogical/behavioural dimension of exercise therapy (e.g. the principle of experience orientation; the principles of structured support of self-responsibility; the principle of simultaneous training, teaching, and experience; the principle of reflexivity; the principle of individuality) (see [34]). Although some topics were discussed in all six focus groups, such as ‘fun and joy in exercise and physical activity,’ it became clear that the topic of PAP is addressed with a high level of variability and heterogeneity by the participants. This is consistent with our recently conducted national survey, which highlighted the importance of the topic of PAP for exercise therapy departments on the institutional level [22]. Previous results from our national survey showed that exercise therapy departments in Germany can be characterized by six typical concepts (e.g. concepts with a focus on physical functioning, or on positive experiences, or on movement practice) with a different level of significance of PAP [23]. Our results demonstrate that exercise therapists feel responsible for PAP and have a corresponding didactic–methodical basic understanding for PAP.

However, exercise therapists in our study rarely referred to scientific theories of behavioural change in their argumentation patterns for PAP. References to existing concepts of theory-based intervention for PAP in exercise therapy [9, 30, 35, 36] were also rarely made by the therapists. Related to this and in comparison to the online survey conducted by Kunstler et al. [15] in Australia, our participants mention only few concrete content and methods for PAP. The majority of the therapists discuss the content in a rather pragmatic and general manner compared to the scientifically discussed approaches and ideas for PAP. This is briefly illustrated by the most frequently discussed topic – namely, ‘joy and fun.’ For this topic, it is almost exclusively argued that a wide range of PA intervention content enables rehabilitants to find something suitable and enjoyable. In contrast to this, scientists’ discussions regarding ‘joy and fun’ include the intensity of the load [37], the possibility of choosing exercises on your own [38], the role of PA-related affect regulation [39], and the systematic adaptation of the exercise content to one’s own motives [40]. These detailed factors do not play a role in the discussion of our focus-group participants.

There may be multiple reasons for this high variability in PAP and the lack of scientific references. First, our sampling was deliberately designed to achieve heterogeneity in participants and maximise the diversity of named concepts. For this purpose, 58 exercise therapists from clinics with six different typical exercise therapy concepts [23] were invited, thus leading to different perspectives regarding the priorities and goals of PAP. Second, in general, PAP is not historically established and is a relatively young topic for exercise therapy. Related frameworks [1, 2] and concepts mentioned above for PAP in exercise therapy were only developed and published during this century. A highly quoted statement claims that it takes 17 years to apply 14% of research evidence to reach clinical practice [41]. As translation needs time, perhaps the current status quo is not so unusual. Third, curricula for therapeutic training and the practical work of therapists are still heavily influenced by the functional biomedical model, which primarily aims at improving bodily functions and thereby neglects psychological and social factors of the rehabilitants [42, 43]. The development and research of behavioural clinical reasoning models [44–46] and the systematic integration of PAP into the training of exercise professions [47] is only recently taking place. Accordingly, bio–psycho–social approaches and the topic of behavioural change comprise new territory for many therapists [48]. Even if some studies prove that physical therapists have the skills and abilities to promote PA [49, 50], therapists have very different knowledge bases, skills, and abilities for a targeted and evidence-based PAP. Fourth, in line with the results of Lowe et al. [18] for the UK, there appears to be no defined common framework and no uniform concept for PAP in German rehabilitation settings. In Germany, there exist several intervention concepts for PAP [9, 30, 35, 36]. However, these have only been acknowledged in a few clinics within the practice of exercise therapy. An exception is the MoVo-LISA concept [30], which is characterized among other things by a published manual. The few exercise therapists who use the MoVo-LISA concept do not implement it on a one-to-one basis; rather, they adapt it to the conditions of their institution. For these adaptations, therapists need skills and abilities that enable them to make targeted choices. Consequently, training concepts should equip therapists with these necessary skills. The development of a framework concept for PAP must be flexible and allow for simple adaptations according to the different conditions of the clinics.

The barriers and facilitators of PAP mentioned by the therapists in our study only partly coincide with those of other studies. Similar to the results of Lowe et al. [18] and Freene et al. [51], the topic of one’s own behaviour as a therapist is only addressed as a promoting factor; lack of self-confidence, a low level of knowledge, or a lack of methodical–didactic skills (e.g. conversation skills) mentioned in other studies [16, 48, 52, 53] were not mentioned by the therapists in our study. This is somewhat surprising given that PAP is difficult to implement and the capability and skills required among therapists are complex [12] but rarely addressed in their current training. However, some of the focus groups expressed the desire for further training on the topic of PAP. As seen with Frawley et al. [54], patient-related factors (e.g. old age, low motivation) are discussed by our participants as negative but not obstructive. Cooperation and communication in the interdisciplinary rehabilitation team is a new topic in our study that was barely addressed in previous research. Our results show that PAP is a joint task of the entire rehabilitation team. From the point of view of exercise therapists, PAP works particularly well when a common language is spoken within the team; PAP is less effective when medical dominance makes it difficult to apply the appropriate exercise therapies.

Our results shed light on the therapists’ content–conceptual and didactic–methodological frameworks for PAP. Thereby, the results have implications for optimising PAP in exercise therapy. First, the discrepancy between scientific evidence and actual practice points to a general implementation problem. In order to improve PAP in therapeutic exercise practice, researchers must systematically disseminate their evidence-based knowledge. A systematic exchange of knowledge between practice and research/science may have the potential to stimulate knowledge, further education, and improved training concepts. What will be needed in the future to “vitalize practice through research’ [32], however, is a roadmap that describes how existing evidence is actually used and implemented by practitioners to improve the quality of therapy. Second, knowledge translation takes place not only from research to practice, but also in the other direction, from practice to research. The generated overview of PAP is able to ‘vitalize research through practice’ [32]. Third, exercise therapy departments have structurally organised content and methods [23], and individual therapists have very different starting conditions – this must be taken into account in comprehensive strategies for the development of PAP in exercise therapy.

One strength of this study is the stratified sampling technique, which was designed to ensure the heterogeneity of participants and maximise the diversity of named concepts. The sampling and the high number of therapists ensure that the participants of the focus groups reflect the spectrum of typical conceptual orientations of exercise therapy in Germany. Thus, the results of the focus groups are likely generalisable for the entire setting of German rehabilitation. The extent to which the results can be transferred to other countries is unclear, as the differences in the training of exercise therapists and the framework conditions of rehabilitation have to be considered.

Our study has weaknesses that need to be addressed. When interpreting the focus-group results, it should be noted that individuals who have certain expertise in PAP are likely to be more active within the focus-group discussions. Nevertheless, the moderators attempted to specifically address people who were not involved in the discussion and thus encouraged them to participate. As a result, the content and methods as well as the barriers and promoting factors of people with less expertise may be less well-represented. Another weakness is the different therapeutic experiences of the participating researchers. The researchers responsible for the coding had more experience and knowledge in the field of orthopaedics *back pain* compared to orthopaedics *total hip/knee replacement*. A consequence of this different expertise could be the still good but lower similarities in the intercoder agreement in the focus group orthopaedics *total hip/knee replacement*.

## Conclusion

In sum, our results prove that exercise therapists are concerned with PAP and use different intervention approaches and ideas to address the topic. Sometimes therapists have clear methodological–didactic action orientations in relation to PAP. Nevertheless, the views of exercise therapy practitioners in many areas of PAP are clearly lagging behind the degree of structuring and complexity currently being discussed in research and included in the available interventional concepts for PAP. These results underline the need for a systematic knowledge transfer from research to practice. The results will help practitioners and researchers to structure and implement high-quality PAP in exercise therapy care.

## Data Availability

For data protection reasons, the data may not be passed on to third parties.

## Abbreviations

KTL: Klassifikation Therapeutischer Leistungen (classification of therapeutic interventions)
PA: Physical activity
PAP: Physical activity promotion
